# Psychological Follow-up During Prenatal Care of Pregnant Women: Insights During the covid-19 pandemic

**DOI:** 10.1055/s-0040-1718451

**Published:** 2021-01-29

**Authors:** Magda Spinello Consul Da Silva, Edward Araujo Júnior, Julio Elito Júnior

**Affiliations:** 1 Escola Paulista de Medicina, Universidade Federal de São Paulo, São Paulo, SP, Brazil

Dear Editor,

The puerperal pregnancy cycle is considered a period of crisis marked by emotional changes in women and men, and may evolve healthily or present psychological disorders that will compromise psychological, physical and marital health. Psychological prenatal care is the monitoring of the pregnant and/or of the couple during pregnancy, the postpartum period and the puerperium until at least the 3 ^rd^ month. The follow-up is for any and all pregnancies in obstetric physiological, pathological, fetal medicine and pregnancy losses. The main objective of psychological support involves preparing the couple to experience motherhood and fatherhood, to develop an active posture of the pregnant and the father, the strengthening of the marital relationship, the facilitation of the maternal-paternal-filial bond and the prevention of postpartum depression and puerperal psychosis. 1

As for the specific objectives, these will be elaborated according to the physical emotional experiences of each patient's pregnancy. It is a personalized interdisciplinary psychological monitoring that aims to provide humanized assistance to women at such a special time and with so many changes. 2 Pregnancy is contemplated by different feelings and transformations each quarter, which means that the stages of psychological prenatal care are adapted to each moment of the evolution of pregnancy. The use of an initial interview and the investigation through psychological anamnesis are important instruments for the knowledge of the psychic functioning of the pregnant woman and the couple. Anguish and anxiety mark the gestational and postpartum 1 ^st^ quarter. Stimulating the free expression of these feelings is one of the most important aspects to be highlighted for facilitating the experience of the puerperal pregnancy cycle. The need for information regarding physical and emotional changes, fetal development, childbirth, neonatal care and breastfeeding are extremely relevant during the approach with the pregnant woman. During psychological counselling, it is important to encourage appropriate body activities according to each case. Meditation, massage and abdominal breathing are also tools taught to the woman and the couple to encourage motherhood, facilitating the moment of delivery and favoring the emotional strengthening of parents with the unborn child. 3

Pregnancy is the pinnacle of a female psychosexual development. The changes that may occur are inherent to the puerperal pregnancy cycle, for both men and women; they lead to increased expectations and anxiety, feelings that can be aggravated during this Covid-19 pandemic period. 4 5 Social distance is mandatory in the midst of the need for health care to the future parents; however, considering the technological advances that have been happening in the last decades, psychological prenatal care remains viable through the use of videoconferencing applications and software such as WhatsApp, Skype and Zoom. 6

Videoconferences allow the obstetric psychologist to perform the service in all its completeness, and the only difference is in the adaptation of some dynamics - visual resources such as video tutorials or illustrated documents can be used to assist the pregnant woman and the couple in performing physical exercises, massages and meditation, for instance. The current moment sets a great challenge in the hands of professionals, who suddenly see themselves in the need of finding new protocols to reinvent the way of dealing with patients. With the use of virtual resources, obstetric psychology sees itself as an opportunity to experience a new way of guiding pregnant women during the day of setting up their maternity.
